# Cytokine responses to *Schistosoma haematobium *in a Zimbabwean population: contrasting profiles for IFN-γ, IL-4, IL-5 and IL-10 with age

**DOI:** 10.1186/1471-2334-7-139

**Published:** 2007-11-28

**Authors:** Francisca Mutapi, Georgina Winborn, Nicholas Midzi, Matthew Taylor, Takafira Mduluza, Rick M Maizels

**Affiliations:** 1 Institute of Immunology & Infection Research, School of Biological Sciences, University of Edinburgh, Ashworth Laboratories, King's Buildings, West Mains Rd, Edinburgh, EH9 3JT, UK; 2 National Institute of Health Research, Box CY 570, Causeway, Harare, Zimbabwe; 3 Department of Biochemistry, University of Zimbabwe, P.O. Box 167, Mount Pleasant, Harare, Zimbabwe

## Abstract

**Background:**

The rate of development of parasite-specific immune responses can be studied by following their age profiles in exposed and infected hosts. This study determined the cytokine-age profiles of Zimbabweans resident in a *Schistosoma haematobium *endemic area and further investigated the relationship between the cytokine responses and infection intensity.

**Methods:**

Schistosome adult worm antigen-specific IFN-γ, IL-4, IL-5 and IL-10 cytokine responses elicited from whole blood cultures were studied in 190 Zimbabweans exposed to *S. haematobium *infection (aged 6 to 40 years old). The cytokines were measured using capture ELISAs and the data thus obtained together with *S. haematobium *egg count data from urine assays were analysed using a combination of parametric and nonparametric statistical approaches.

**Results:**

Age profiles of schistosome infection in the study population showed that infection rose to peak in childhood (11–12 years) followed by a sharp decline in infection intensity while prevalence fell more gradually. Mean infection intensity was 37 eggs/10 ml urine (SE 6.19 eggs/10 ml urine) while infection prevalence was 54.7%. Measurements of parasite-specific cytokine responses showed that IL-4, IL-5 and IL-10 but not IFN-γ followed distinct age-profiles. Parasite-specific IL-10 production developed early, peaking in the youngest age group and declining thereafter; while IL-4 and IL-5 responses were slower to develop with a later peak. High IL-10 producers were likely to be egg positive with IL-10 production increasing with increasing infection intensity. Furthermore people producing high levels of IL-10 produced little or no IL-5, suggesting that IL-10 may be involved in the regulation of IL-5 levels. IL-4 and IFN-γ did not show a significant relationship with infection status or intensity and were positively associated with each other.

**Conclusion:**

Taken together, these results show that the IL-10 responses develop early compared to the IL-5 response and may be down-modulating immunopathological responses that occur during the early phase of infection. The results further support current suggestions that the Th1/Th2 dichotomy does not sufficiently explain susceptibility or resistance to schistosome infection.

## Background

Schistosomiasis is a major human parasitic disease caused by trematode parasites of the genus *Schistosoma*. Of the three major human schistosome species, *S. haematobium*, causing urinary schistosomiasis, is the most prevalent species in sub-Saharan Africa where it is responsible for a substantial amount of schistosome-associated pathology [1].

The role of acquired immunity in reducing schistosome infection intensity in human populations has been subject to intense analysis [2-7]. Schistosome immuno-epidemiology studies have shown that the development of antigen-specific immune responses is related to cumulative exposure to parasite antigens [8,9] and that the rate of development of different components of these responses give distinct profiles across the host age range [10]. These profiles have facilitated the identification of responses associated with protection to infection/re-infection. For example, anti-worm IgE levels were shown to increase with age in *S. haematobium *exposed/infected children in the Gambia, while infection intensity declined [11] and the combined change in IgA (declining) and IgG1 (rising) with age in Zimbabwean populations residing in *S. haematobium *endemic areas was associated with resistance to infection [12]. Thus an understanding of the rate of development of parasite-specific immune responses derived from age profiles is useful in interpreting susceptibility and resistance to infection as well as the development of pathology. This is particularly important for the cellular responses, which determine the majority of effector functions and immune mediated schistosome pathology [13-15]. Detailed studies following the development of schistosome-specific cellular responses over a period of time have been conducted in the mouse model, but such studies have yet to be conducted in human schistosomiasis (see [14] for review). Given that differences occur in the immunology and immunopathology of murine and human schistosomiasis [16,17], human studies are essential for a clearer definition of human schistosome immuno-epidemiology.

Mouse experimental schistosomiasis studies suggest that the development of effector Th1/Th2 and immuno-modulatory responses reflect the parasite's developmental stage (see [14] for review) so that Th1 responses predominate in the early acute phase followed by the emergence of Th2 responses (stimulated by egg antigens) and decrease in Th1 responses (down-modulated through an IL-10-dependent mechanism) [18]. To date, detailed studies giving an indication of the time course of the development of cellular responses against the parasite have not been performed in human schistosomiasis. Thus this study aims to determine the cytokine-age profiles of Zimbabweans resident in *S. haematobium *endemic areas. The cytokines to be studied are IFN-γ, a marker for Th1 responses, IL-4 and IL-5, markers for Th2 responses, and IL-10, originally classified as both a Th1 and Th2 cytokine in humans [19] but now also seen as a marker for immuno-modulatory (including regulatory responses) [18,20,21].

The study also determines the relationship between these cytokine responses and infection intensity. Previous studies have suggested that anti-helminth immune responses fall into a clear Th1 (pro-inflammatory) and Th2 (anti-inflammatory) dichotomy with resistance to schistosome infection being associated with Th2 responses [22-25]. More recently there has been mounting evidence that unlike the intracellular pathogens such as *Toxoplasma gondii *and *Leishmania major *where there is a clear polarisation of the protective response to Th1, the relationship between the Th1/Th2 dichotomy to the development of acquired immunity against schistosomes and other helminths remains unclear ([26-30], see [31] for review). For example, we have previously shown that anti-egg Th1 and Th2 cytokine responses in *S. haematobium *infected/exposed Zimbabwean children did not show a clear pattern with infection intensity [32]. In fact, the Th2 cytokines IL-4 and IL-5 gave contradictory results [32,33]. Our studies and those of others have suggested that some of the differences reported on the correlation between Th1/Th2 responses may be related to differences between schistosome species or to antigens used and/or histories of infection in diverse epidemiological settings [34]. However the differences may be a refection of the complexity of the relationship between cytokine data and infection intensity which goes beyond the Th1/Th2 dichotomy. We therefore investigated the relationship between the different cytokines (through correlation and data reduction methods) and used the results from a principal component analysis to relate infection intensity to the cytokine data after describing the epidemiology of the infection in the population.

## Methods

### Parasite material

Lyophilised soluble *S. haematobium *adult worm antigen (SWAP) was obtained from the Theodor Bilharz Institute (Egypt) and reconstituted as previously described elsewhere [35]. The parasite strain is one used for previous immuno-epidemiology studies [10].

### Study subjects

The study was conducted in the Mashonaland East Province of Zimbabwe (31°30'E; 17°45'S) where *S. haematobium *is endemic. The study area is described in detail elsewhere [36] and the participants have been participating in an ongoing study of the immunoepidemiology of human schistosomiasis [35,37]. Permission to conduct the work in this province was obtained from the Provincial Medical Director (PMD) while ethical approval was received from the Medical Research Council of Zimbabwe. Following explanation of the study aims and procedures to the community, school children and their teachers, informed consent was obtained from participants or their parents/guardians. The villages were selected because health surveys regularly conducted in the region by the PMD showed little or no infection with other helminths and a low *S. mansoni *prevalence (<5%). Prior to our study the selected villages had not been included in the National Schistosome Control Programme (run by the Ministry of Health and Child Welfare in Zimbabwe) and therefore had not received treatment for schistosomiasis or other helminth infections meaning that we could study natural immune responses in the absence of drug-altered schistosome responses [12,38]. The main activity in these villages is subsistence farming and human water contact is frequent with at least 4 contacts/person/week due to insufficient safe water and sanitation facilities (see [36] for studies in neighbouring villages). Drinking water is collected from open wells while bathing and washing is conducted in two main rivers in the villages. Most families maintain a garden located near the river where water is collected for watering the crops. The schools surveyed (a secondary school and its feeder primary school (i.e. where the majority of the primary school children come from) Goromonzi and Shangure Schools in Goromonzi Village, and Chindenga and Nyambanje Schools in Mutoko Village) were all in close proximity to rivers. Stool and urine specimens were assayed for *S. haematobium, S. mansoni *and geohelminths using standard procedures [39,40]. In order to be included in the study, participants had to meet all the following criteria: 1) have provided at least 2 urine and 2 stool samples on consecutive days; 2) be negative for intestinal helminths including *S. mansoni *(no one was excluded on this criteria as everyone was negative for these infections), 3) have given a blood sample. 190 participants aged 6–40 years met these criteria and formed our study population. As is standard in all our studies after collection of all samples, all participants were offered treatment with the recommended dose of praziquantel (40 mg/kg of body weight).

### Cytokine stimulations and assays

Whole blood stimulations were set up following a protocol developed at the University of Cambridge, UK [41]. 10 mls of heparinised venous blood were collected and immediately diluted 1:4 in RPMI 1640 medium (Sigma) with penicillin (50 U/ml), streptomycin (50 μg/ml), L-glutamine (2 mM) (Sigma) and 10% AB serum. 250 μl of this media was added to a 48-well culture plate which already contained 25 μl of antigen in RPMI media described above at 10 μg/ml (SWAP, or Con A) or media alone. The study focused on responses against whole worm antigen to provide a complete picture of antibody and cellular responses against worm antigens (see [35,37] presenting results of antibody responses of participants from this population).

Additional cultures were set up to detect the effect of neutralising IL-10 using samples from a subgroup of the participants who had provided enough blood to allow additional assays (n = 17, age range 6–17 years, mean infection intensity range 0–111 eggs/10 ml urine). For these cultures, 50 μl/well of 1 μg/ml anti-human IL-10 (rat IgG1 anti-human, JES3-9D7) antibody was added to half the plate before addition of the whole blood and 50 μl/well of 1 μg/ml isotype control (rat IgG1 R3-34) was added to the remaining half, so that the same sample was run on the same plate in wells containing anti-IL-10 antibody and the isotype control. The plates were placed in a glass jar, together with a gas generating kit (Oxoid) and incubated at 37°C for 48 hrs. Supernatants were harvested into cryotubes and stored at -20°C in the field prior to transportation at -20°C to the United Kingdom for analysis. Samples were thawed and all 4 cytokines assayed at the same time for each sample. IFN-γ, IL-4, IL-5, IL10 cytokine assays were conducted using capture ELISAs with antibody sets from BD Pharmigen following previously published protocols [41]. All assays were conducted in duplicate.

### Statistical analyses

Initial analyses were to determine the relationship between cytokine levels and infection intensity, as well as between cytokine pairs. After exploratory plots showed that these relationships were non linear and were directional (either positive or negative), the analyses were conducted using a one-tailed non parametric Spearman correlation procedure [42].

The second step was to determine if the cytokines followed a distinct age profile. Because of the potential confounding relationships of infection intensity, host age and sex on cytokine levels (see [43]), the effect of infection intensity and sex on net cytokine level was allowed for before testing if age had a significant association with net cytokine level. This was achieved by a multivariate analysis of variance (MANOVA). The dependent and independent variables were transformed or categorised as appropriate to satisfy the assumptions of the parametric tests. Cytokine level (square root transformed) was the dependent variable and sex (2 categories), age (6 categories as shown in Table 1) and infection intensity (log_10 _(x+1) transformed) were the independent variables.

**Table 1:** Description of study population

**Age group (years)**	**Male (sample size)**	**Female (sample size)**	**Total (sample size)**	**Range of egg counts/10 ml urine**
6–10	17	12	29	0–465
11–12	14	17	31	0–535
13–14	18	22	40	0–362
15–16	17	16	33	0–327
17–18	14	9	23	0–168
19+	7	27	34	0–123

**Figure 1:**
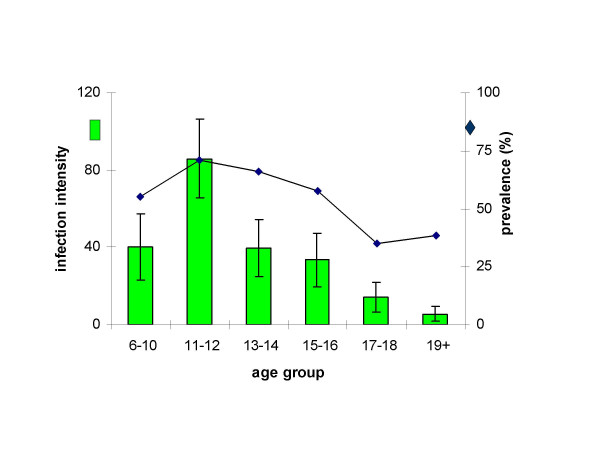
Age-infection profile of the study population. The histograms represent mean infection intensity for each age group calculated using a mean of 3 urine samples/person and bars represent the standard error of the mean. The line graph represents infection prevalence. Sample sizes are given in Table 1.

The table gives the sample sizes in each of the age groups represented in Figure 1.

The next statistical tests were to determine the relationship of the cytokine responses (cumulatively) and infection status. This was achieved by factor analysis using principal components (PCA) [42]. PCA is a standard technique for reducing multivariate data down to its main independent features [42]. This procedure was used in this instance for two reasons (1) to avoid type I and type II errors in multiple tests using correlated independent variables [43,44] and (2) to capture the effects of all cytokines in a single analysis since the responses are likely to be acting simultaneously (see [45]). Following the factor analysis principal components were analyzed further with logistic regression to determine the risk factors associated with being infected (egg positive) or uninfected (egg negative). Independent variables included the principal components, sex and age group (categorised as above).

The final statistical test was a two-tailed paired t-test to determine the effect of blocking IL-10 on cytokine levels conducted on the square root transformed cytokine data. All statistical tests were conducted using the software package SPSS. There is an outlier in the IL-4 data, therefore all statistical procedures were repeated with the single outlier removed; this did not affect the outcome of any of the statistical tests, so the results reported here include the outlier. All significant p values (<0.05) were subjected to Bonferoni testing [46].

## Results

### Prevalence and intensity of infection

Mean infection intensity for the study population was 37 eggs/10 ml urine with a SE of the mean of 6.19 eggs/10 ml urine (range 0 eggs – 535 eggs/10 ml urine) while infection prevalence was 54.7%. Infection prevalence was similar across all age groups except in children aged 13–14 and 17–18 where males had higher prevalences than females. Although these infection levels are moderate relative to reports from other areas in Zimbabwe [10], the World Health Organisation denotes this prevalence level as high and infection intensity also as high as defined by having more than 10% of the population with >50 eggs/10 ml of urine (36 of the 190 participants had >50 eggs per 10 ml urine) [47]. Infection rose to peak in childhood (11–12 years) followed by a sharp decline in infection intensity while prevalence fell more gradually as shown in Figure 1. Infection levels in this population peaked at lower levels and in older children compared to high infection areas in Zimbabwe [10]. There were no other helminth species detected in the participants enrolled for this study.

### S. haematobium antigen specific cytokine responses – age profiles

**Figure 2:**
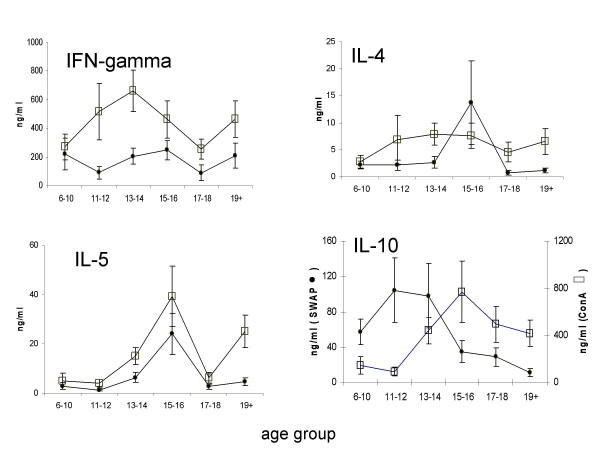
Age-cytokine profiles for IFN-γ, IL-4, IL-5 and IL-10. Means (net values after subtracting corresponding media values for each individual) for each age group are plotted with bars representing standard errors of the mean. Squares represent responses to ConA stimulation, and circles represent parasite-specific responses.

We measured levels of IFN-γ, IL-4, IL-5 and IL-10 in vitro responses to mitogen (ConA) and parasite adult worm antigen (SWAP) challenge. In general, responses to ConA were higher than those against parasite antigens and spontaneous cytokine production was lower than parasite and mitogen-induced responses. Of the 190 participants, the following produced detectable parasite specific cytokines; 114 (60%) produced IFN-γ, 86 (45%) produced IL-4, 103 (54%) produced IL-5 and 144 (76%) produced IL-10, while 32 people (17%) produced all 4 cytokines simultaneously. The most distinct difference between age profiles of mitogen and parasite-specific responses was in IL-10 as shown in Figure 2. Levels of IL-10 rose to peak with infection intensity in 11–12 year olds, while levels of IL-4 and IL-5 rose more slowly peaking later in 15–16 year olds.

In order to compare age profiles we had to determine if the cytokines produced varied with host age after allowing for the effects of sex and infection intensity. MANOVA analyses showed that host age significantly affected levels of parasite-specific IL-4 and IL-5 and IL-10 but not IFN-γ as shown in Table 2.

**Table 2:** Output of from the multi-variate analysis of variance conducted on all study participants

**Cytokine**	**Age group**
IFN-γ	F = 1.532, df = 5,190, p = 0.182
IL-4	F = 3.277, df = 5,190, **p = 0.007**
IL-5	F = 4.875, df = 5,190, **p < 0.001**
IL-10	F = 2.465, df = 5,190, **p = 0.034**

The analysis determining if cytokine levels varied with host age thus testing whether the patterns represented in Figure 2 are significant. The statistical procedure allowed for the effects of sex and infection intensity on net cytokine level before testing if age had a significant effect on net cytokine level. F-value represents the F-test statistic from the MANOVA, df = degrees of freedom. Significant p-values are in bold.

### Single cytokine patterns with infection intensity

**Figure 3:**
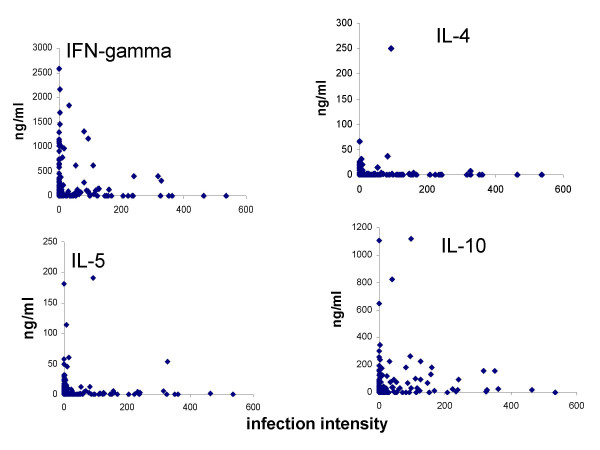
Relationship between net parasite-specific cytokine level (ng/ml) and infection intensity (eggs/10 ml urine).

Plots of the relationship between cytokine levels and infection intensity (Figure 3) indicate that

IFN-γ and IL-4 production did not show significant associations with infection intensity. People carrying high levels of infection (>50 eggs/10 ml urine) tended to make low levels of IL-5 (<10 ng/ml), while people with higher IL-5 levels had low levels of infection although this relationship was not statistically significant. IL-10 levels were significantly correlated to infection intensity and this correlation was positive as shown in Table 3.

**Table 3:** Relationships between cytokine pairs and between cytokines and infection intensity

	**Infection intensity**	**IFN-γ**	**IL-4**	**IL-5**
**Infection intensity**	-			
**IFN-γ**	-0.003	-		
**IL-4**	-0.039	0.287**	-	
**IL-5**	0.025	0.352**	0.494**	
**IL-10**	0.197**	0.040	0.093	-0.169**

**Figure 4:**
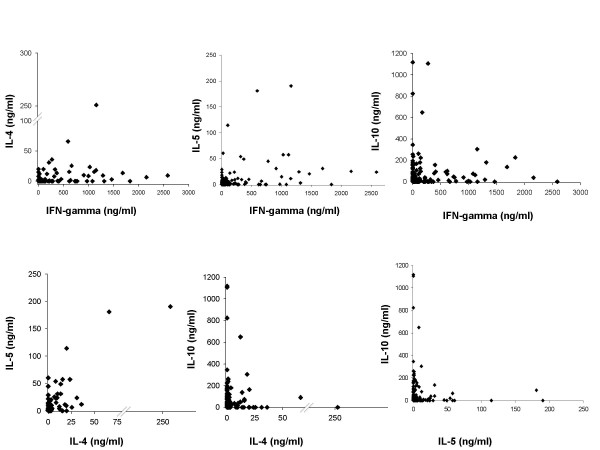
Relationship between pairs of cytokines (net parasite-specific) (ng/ml) showing the positive correlation between all cytokines except IL-10, which when paired with IL-5, shows a negative correlation.

The statistics are from a one tailed non parametric Spearman test. This analysis determined whether the relationships in Figure 3 and Figure 4 are significant. ** represents correlations were p = 0.01.

### Relationship between cytokine levels and infection status

The relationship between infection status (egg positive or negative) and cytokine response was explored using factor analysis and logistic regression. The factor analysis approach was essential in order to reduce the cytokine data to fewer variables thus minimising problems associated with multicolinearity [44] as well as to produce variables containing all the cytokines measured. Factor analysis gave 2 principal components with the strongest contributing variables being IFN-γ, IL-4, IL-5 for PCA1 and IL-10 for PCA2. The amount of variation explained by each of the components is given in Table 4 while the weightings of each cytokine in the two components is given in Table 5. Thus the strongest dichotomy in the immune responses was between the effector T cell cytokines in PCA1 and the immuno-modulatory cytokine IL-10 in PCA2. Logistic regression analyses showed that infection status was significantly affected by PCA 2 (made up largely of IL-10) (p = 0.039, β = 0.51 with a SE of 0.247, -2 Log likelihood of 6.522) and that the effect was positive. Therefore people producing high levels of parasite-specific IL-10 were significantly more likely to be egg positive than those producing lower levels of the cytokine.

**Table 4:** Variance explained by the principal components extracted from the cytokine data.

**Component**	**Initial eigenvalues**		
	**Total**	**% variance**	**Cumulative %**

1	1.96	49.07	49.07
2	1.018	25.56	74.53
3	0.782	19.55	94.08
4	0.237	5.92	100

There were 4 principal components extracted from the data. The percentage of variance in the cytokine data explained by each of the components is given both individually and cumulatively showing that the first 2 components explained the greatest percentages.

**Table 5:** Weighting (coefficient) of each cytokine in the values of the two principal components

**Cytokine**	**Principal component 1**	**Principal component 2**
IFN-γ	**0.583**	0.270
IL-4	**0.888**	-0.054
IL-5	**0.912**	0.072
IL-10	-0.046	**0.968**

The principal components were extracted by the factor analysis of cytokine responses. Strong loadings (> 0.5) are indicated in bold. This procedure involves a mathematical procedure that transforms a number of correlated variables into a smaller number of uncorrelated variables called principal components. The first principal component accounts for as much of the variability in the data as possible, and each succeeding component accounts for as much of the remaining variability as possible [42]. This procedure is valuable for this immunology data because it allows the simultaneous effects of all the cytokines to be studied at the same time which is a better reflection of the biological situation that the standard procedure of studying correlations between individual cytokines and infection intensity [43]. The two principal components were then used to determine the cumulative effects of the cytokine responses on infection status by logistic regression.

### Correlations between cytokines

The relationships between pairs of cytokine are plotted in Figure 4 and the results from the statistical tests are shown in Table 3. The most notable correlation was between parasite specific IL-10 and IL-5, which was significantly negative. Other cytokines which were significantly correlated were INF-y with both IL-4 and IL-5 and IL-4 and IL-5, which were all positively correlated. IL-4 and Il-10 showed no significant relationship with each other. People produced high levels of either IL-5 (>12 ng/ml) or IL-10 (>140 ng/ml) but not high levels of both. These significant positive correlations between INF-y, IL4 and IL-5 and the negative correlation between IL-10 and IL-5 indicate the relationships between the cytokines which underlie the PCA analyses.

### Effect of blocking IL-10 on levels of IFN-γ, IL-4 and IL-5

Antibody to IL-10 was added to in vitro cultures during parasite antigen challenge. Blocking IL-10 in this way resulted in a significant change only in the level of parasite-specific IL-5, which increased from a mean of 5.15 ng/ml (SE = 1.39) in the control samples incubated in the presence of an isotype control to 6.56 ng/ml (SE 1.30) in the samples incubated in the presence of anti-IL-10 antibodies (T-Value = 3.12, df = 16, p = 0.007). IFN-γ and IL-4 production did not differ significantly between IL-10 blocked and normal control samples (T = 0.07, df = 16, p = 0.948; T = -0.62, df = 16, p = 0.542 respectively).

## Discussion

This study followed the development of naturally acquired schistosome-specific cytokine responses with host age to determine the rate of development of these responses. We have previously compared the same cytokine response across populations exposed to different infection intensities and showed that the rate of development of *S. haematobium*-specific cytokine responses was affected by the host's history of infection, with responses developing earlier in areas of high infection intensity compared to areas of low infection intensity [48]. Here we compared the profiles of different cytokine responses within one host population. The cytokines studied were IFN-γ, a marker for Th1 responses, IL-4 and IL-5, markers for Th2 responses, and IL-10, originally classified as both a Th1 and Th2 cytokine in humans [19] but now also seen as a marker for immuno-modulatory responses including regulatory T cell responses [18,20,21].

In our study the different cytokines followed distinct age-profiles with the relationship between age and cytokine level being significant for IL-4 and IL-5 cytokines believed to be important in protection against re-infection with *S. haematobium *infection [22] as well as IL-10 which has previously been associated with immunomodulation. Levels of IL-10 response rose to peak early in childhood declining thereafter while levels of IL-4 and IL-5 rose more slowly to peak later with the peak in IL-4 being less pronounced than that of IL-5 so that the age groups where infection intensity and prevalence are lowest coincide with high IL-5 and low IL-10. Furthermore the study showed a positive correlation between IL-10 and infection intensity which both rose to peak in children aged 11–12 years and declined thereafter. The positive association of IL-10 and infection intensity has previously been reported in *S. mansoni*-exposed Brazilians [34]. Two possible explanations for this positive association are, (1) IL-10 could simply be reflecting exposure to parasite antigens or (2), high levels of IL-10 are stimulated by high infection levels so as to prevent the development of excessive Th2-mediated pathology in addition to Th1-mediated pathology [14,26]. The latter is consistent with earlier immuno-epidemiological studies of *S. haematobium *infection reporting immuno-suppression of schistosome responses in children [49,50] and Th2 responses in adults with little or no infection [48]. In the mouse model IL-10 reduces schistosome related liver damage and prolongs host survival [18], both of which are Th2-mediated, and in human *S. haematobium *and *S. mansoni *infections high IL-10 production has been associated with reduced schistosome-induced pathology(see [17] for review).

There was no correlation between infection intensity and either IL-4 or IL-5. A previous study in a *S. mansoni *endemic Ugandan fishing community suggested that high IL-5 levels are associated with low *S. mansoni *levels in older individuals since high levels coincided with the lowest infection levels [41]. However, the Ugandan study showed no significant relationship between current infection intensity and IL-5 levels. This result is similar to our current observation that there was no significant correlation between these two parameters. Our study and that from the Ugandan fishing community support suggestions that IL-5-mediated responses against helminths may be directed against incoming parasites rather than existing worm burdens [51,52]. This might also explain the lag between the age profiles of IL-5 levels and infection intensity we observed in this study.

When the relationship between all the cellular responses in our study was investigated by factor analysis, responses were divided into two principal components, i.e. IFN-γ/IL-4/IL-5 vs. IL-10 dichotomy rather than the more conventional Th1 vs. Th2 dichotomy [22,48,53]. Thus this analysis divided the cytokine responses into the Th1/Th2 effector responses and the immuno-modulatory IL-10 response. Further analysis of how this dichotomy related to infection level showed that while the relationship between the first component and infection level was unclear, people producing high levels of parasite-specific IL-10 (represented by the second PCA) being significantly more likely to be egg positive than those producing lower levels of the cytokine. These results suggest that the Th1/Th2 dichotomy may not sufficiently describe the development of resistance against schistosomiasis which, is consistent with previous schistosomiasis studies which have reported mixed Th1/Th2 responses against adult worm antigens [41] and current suggestions that the outcome of helminth infection is related to the balance between effector (Th1/Th2) and regulatory responses [54]. The IL-10 response in this population was negatively correlated with the IL-5 response and blocking IL-10 resulted in an increase in parasite-specific IL-5 produced. Taken together these results support the proposition that in schistosomiasis at least, IL-10 is acting as a regulatory cytokine which inhibits a protective IL-5 dependent response [3,17,18,55]. Various cells have been implicated in IL-10 production [26,56] but the distinct cellular sources of this cytokine remain poorly defined in both human and experimental schistosomiasis. We are therefore currently determining the source of the parasite-specific IL-10 measured in this study.

## Conclusion

Taken together, our results show first that the IL-10 response occurs early, peaking in young children, suggesting that immuno-modulatory responses are already present in the young age groups indicating a parallel to the mouse model of schistosomiasis in which IL-10 down-modulates the early phase of infection [14]. Of course such parallels have to be interpreted with caution as human studies differ from experimental models in many respects not least by the lack of a precise measure of the point of infection in an endemic population and more so because of the potential for sensitisation to parasite antigens in utero [57,58]. Second, our data indicate that IL-10 may be exerting its modulatory effect by down-regulating IL-5 production. More broadly our results also support current hypotheses suggesting that the balance between effector and immuno-modulatory responses is an important factor in the development of acquired resistance to schistosomes [26,59].

## Competing interests

The author(s) declare that they have no competing interests.

## Authors' contributions

FM conducted the field work, developed and supervised the immunoassays (together with MT), analyzed the data and drafted the manuscript. All these stages received significant contributions from all the other authors as follows: TM and NM conducted field work collecting parasitology and immunology samples from the participants. GW conducted the laboratory assays and conducted the initial statistical analyses. The overall study was overseen by RMM who also helped with the preparation of the manuscript. All authors read and corrected draft copies of the manuscript and approved the final version.

## Pre-publication history

The pre-publication history for this paper can be accessed here:


